# Alcohol ADME in Primates Studied with Positron Emission Tomography

**DOI:** 10.1371/journal.pone.0046676

**Published:** 2012-10-01

**Authors:** Zizhong Li, Youwen Xu, Don Warner, Nora D. Volkow

**Affiliations:** 1 Medical Department, Brookhaven National Laboratory, Upton, New York, United States of America; 2 National Institute on Alcohol Abuse and Alcoholism, Bethesda, Maryland, United States of America; 3 National Institute on Drug Abuse, Bethesda, Maryland, United States of America; National Institute of Health, United States of America

## Abstract

**Background and Purpose:**

The sensitivity to the intoxicating effects of alcohol as well as its adverse medical consequences differ markedly among individuals, which reflects in part differences in alcohol's absorption, distribution, metabolism, and elimination (ADME) properties. The ADME of alcohol in the body and its relationship with alcohol's brain bioavailability, however, is not well understood.

**Experimental Approach:**

The ADME of C-11 labeled alcohol, CH_3_^11^CH_2_OH, **1** and C-11 and deuterium dual labeled alcohol, CH_3_^11^CD_2_OH, **2** in baboons was compared based on the principle that C–D bond is stronger than C–H bond, thus the reaction is slower if C–D bond breaking occurs in a rate-determining metabolic step. The following ADME parameters in peripheral organs and brain were derived from time activity curve (TAC) of positron emission tomography (PET) scans: peak uptake (C_max_); peak uptake time (T_max_), half-life of peak uptake (T_1/2_), the area under the curve (AUC_60min_), and the residue uptake (C_60min_).

**Key Results:**

For **1** the highest uptake occurred in the kidney whereas for **2** it occurred in the liver. A deuterium isotope effect was observed in the kidneys in both animals studied and in the liver of one animal but not the other. The highest uptake for **1** and **2** in the brain was in striatum and cerebellum but **2** had higher uptake than **1** in all brain regions most evidently in thalamus and cingulate. Alcohol's brain uptake was significantly higher when given intravenously than when given orally and also when the animal was pretreated with a pharmacological dose of alcohol.

**Conclusion and Implications:**

The study shows that alcohol metabolism in peripheral organs had a large effect on alcohol's brain bioavailability. This study sets the stage for clinical investigation on how genetics, gender and alcohol abuse affect alcohol's ADME and its relationship to intoxication and medical consequences.

## Introduction

The evaluation of alcohol's ADME in intact animals is complicated by the fact that alcohol and its major metabolites: acetaldehyde, acetate, and carbon dioxide, are small and rapidly diffusible molecules that can penetrate cellular membranes and diffuse within the water volume of the body. In addition, no high affinity non-covalent ethanol binding sites have yet been found. As a result, the binding and bio-distribution assays with radiolabeled ligands that are routinely used in pharmacology research are not suitable for studies of alcohol's ADME.

Enzymatic oxidation of ethanol to acetaldehyde and then to acetic acid is the major ethanol metabolic pathway *in vivo*. Clinical studies suggest that organ damage from excessive alcohol consumption is at least partially related to alcohol metabolic products [1], [2]. Slower acetaldehyde metabolism in Asian populations is responsible for their lower lifetime prevalence of alcohol abuse disorders than in other ethnic groups [3], [4], but it is also responsible for the significantly higher risk of digestive tract cancers among heavy drinkers [5], [6], [7]. Greater brain atrophy [8] and liver and cardiac damage among women alcoholics than among men might be attributed to gender differences in metabolism of alcohol [9], [10], [11], [12]. Similarly differences between men and women in the sensitivity to alcohol's behavioral and central neurological effects are likely to reflect in part differences in alcohol's metabolism by peripheral tissues [8], [13]. Metabolism of alcohol in the brain may also modulate its behavioral effects as shown in rats that the oxidation of alcohol to acetaldehyde by catalases in the Ventral Tegmental Area (VTA) was reported to be essential for alcohol's rewarding effects [14].

Alcohol's elimination in experimental animals has been studied by monitoring blood ethanol concentration and the distribution of stable isotopically and radioisotopically labeled ethanol and its isotopologues [15], [16], [17]. Detailed physical and biochemical transformation data of ethanol in isolated liver [18], lung [19], kidney [20], and brain [21], [22], [23] have been reported. There are, however, few studies on alcohol's whole body regional distribution and pharmacokinetics in intact primates. In cats, studies with carbon-11 labeled alcohol showed high levels of radioactivity in liver, heart and head, which presumably reflected the accumulation of alcohol or its metabolic products in these organs [24].

In humans, the brain imaging studies with magnetic resonance spectroscopy (MRS) showed that ethanol's concentration in the brain plateaus at around 35 min after oral administration of pharmacological doses of ethanol [25]. A brain imaging study with positron emission tomography (PET) and O-15 water (marker of blood flow) and C-11 ethanol suggested that ethanol's distribution in brain was in part mediated by interactions with GABA and/or NMDA receptors [26]. Glutamate, voltage-gated calcium channels, opioid, dopamine, serotonin, and acetylcholine receptors are affected by alcohol, but the interaction of ethanol with these receptors is believed to be transient and of low affinity apparently acting as a “molecular lubricant” that alters the protein function [27].

PET is a tool of choice to study drug distribution in human and experimental animals in real time. The combination of PET and deuterium kinetics isotope effect can be used to differentiate a drug's biochemical mechanism in the organs of interest [28]. The deuterium isotope effect is based on the principle that the C–D bond is stronger than C–H bond, and the reaction will be slower if a C–D bond breaking is involved in a metabolic rate-determining step. The deuterium isotope effect has been used to determine the contribution of various ethanol oxidative metabolic pathways both *in vitro* and *in vivo* [29], [30]. Here we aimed to use C-11 labeled alcohol, **1** and C-11 and deuterium dual labeled alcohol, **2** as a pair of PET tracers to study alcohol ADME in the baboon including its metabolism within the various organs. We also evaluated the relationship between overall alcohol's ADME in the various organs of the body and its bioavailability in the brain. Thus we hypothesize that, in the same subject, we would observe a significant isotope effect in the liver and the kidneys, which are the organs that metabolize alcohol but not in heart and lungs, which don’t metabolize alcohol. We do not expect to observe significant isotope effect in the brain due to its negligible contribution to the overall alcohol metabolism [31], however uptake pattern difference of **1** and **2** in different brain region in the same subject could be indicative of isotope sensitive alcohol metabolism in brain.

## Methods

### The preparation of CH_3_^11^CH_2_OH, 1 and CH_3_^11^CD_2_OH, 2

Tracers **1** and **2** were prepared according to a modified literature procedure [32]. Briefly, ^11^CO_2_ from a target that was trapped in a solution of methyl magnesium bromide (MeMgBr) in ether. The product was then reduced by lithium aluminum hydride or lithium aluminum deuteride, hydrolyzed by sodium hydroxide aqueous solution (5 N) to give the crude **1** or **2** respectively with [^11^C]methanol (3 to 7%) and [2-^11^C]isopropanol (5 to 30%) as the major radiochemical impurities. The reaction mixture was then subjected to HPLC purification using a self-packed fermentation column and water as elute and the purified product was formulated into an injectable saline solution. The radiochemical purity of C-11 ethanol was greater than 99% without detectable chemical and radio-chemical impurities in the solution.

### Baboon Preparation

Baboon studies were approved by the Institutional Animal Care and Use Committee of Brookhaven National Laboratory and the baboons were housed and maintained in an accredited animal facility certified by the Association for Assessment of Laboratory Animal Care. Animals had free access to food, water and toys and were monitored during study by veterinary stuff. PET studies were performed on four female baboons (*Papio Anubis*). Baboons were anesthetized with an intramuscular injection of ketamine hydrochloride (10 mg/kg), intubated, and for studies in which alcohol is administrated orally, a nasogastric tube was installed. The baboon was transported from animal facility to the PET laboratory by a certified veterinary nurse and maintained on a gaseous mixture of oxygen, nitrous oxide, and isoflurane throughout the imaging session. Catheters were placed in an antecubital vein for radiotracer injection and for plasma sampling for radioactive alcohol, acetaldehyde, and acetic acid quantification. EKG, blood pressure, O_2_ saturation, and respiratory setting were continuously monitored throughout the study.

### PET studies

PET scans were performed on a Siemens HR+ high resolution, whole-body PET scanner (63 slices, 4.5×4.5×4.8 mm) in 3-dimensional acquisition mode. Before each scan, a transmission scan was obtained with a ^68^Ge rotating rod source for attenuation correction. For brain imaging, the head of the baboon was positioned at the center of the field as defined by imbedded laser lines with help of a stereotactic head fixation device. The PET measurements were carried out according to the protocols described in Table 1. Data acquisition was started immediately after the injection. The images were summed from 0 min to 59 min for liver, heart and lungs, and from 0 min to 10 min for the brain. For the peripheral organs, circular regions of interests (ROIs) were drawn manually on the heart, lungs, liver and the kidneys on a summed image and projected onto dynamic images to derive TAC. For the brain circular ROI were obtained in the striatum, cerebellum, thalamus, occipital cortex, frontal cortex, temporal cortex, cingulate, global (whole brain), and the white matter. The average radioactivity in the ROIs from each organ was taken as the tracer uptake in that organ. The images were also reconstructed into dynamic images containing 27 continuous slices to derive TAC. The area under the curve of each organ was calculated by the trapezoidal method up to the termination of acquisition (60 min). The baboon had a radial arterial cannula in the wrist to permit continuous counting of blood radioactivity concentration with a bismuth germinate counter during the course of the experiment. Blood samples were also taken after the injection and the activity in the blood sample was counted in a NaI well counter to derive the plasma and the metabolites corrected plasma curves. The radioactive ethanol, aldehyde, and acetic acid were quantified with HPLC on a fermentation column. Acetate, acetaldehyde, and ethanol eluted at 8, 10 and 12 min (elute: 30 mM HCl, flow rate 0.5 mL/min) respectively.

**Table 1:** Summary of Baboon PET studies.

Study name	Name of baboon (BW)	Organ of interesting	Radiotracer	Administration method (dose and formulation)	The scan protocol	
***Brain uptake and deuterium isotope effect***						
BEJ135	Pearl (14 kg)	Brain	1 (first scan) 2 (second scan)	i.v. (75.48 MBq) (76.22 MBq)	4×30 s, 4×60 s, 4×120 s, 9×300 s	
BEJ142	Pearl (14 kg)	Brain	1	i.v. (164.28 MBq)	12×10 s, 8×30 s, 4×120 s, 8×300 s	
BEJ152	Pearl (14 kg)	Brain	2 (first scan) 1 (second scan)	i.v. (145.78 MBq) (145.78 MBq)	12×10 s, 8×30 s, 4×120 s, 8×300 s	
BEJ166	Pearl (15.1 kg)	Brain	2 (first scan) 1 (second scan)	i.v. (42.55 MBq) (140.60 MBq)	12×10 s, 8×30 s, 4×120 s, 8×300 s	
BEJ167	Spicey(17.4 kg)	Brain	2 (first scan) 1 (second scan)	i.v (142.82 MBq) (109.52 MBq)	12×10 s, 8×30 s, 4×120 s, 8×300 s	
BEJ179	Pearl(N/A)	Brain	1 (first scan) 1 (second scan)	i.v (139.12 MBq) (165.02 MBq)	12×10 s, 8×30 s, 4×120 s, 8×300 s	
***Peripheral uptake and deuterium isotope effect***						
BEH131	Missy(15.5 kg)	Torso	2 (first scan) 1 (second scan)	i.v (175.01 MBq) (101.01 MBq)	12×10 s, 8×30 s, 4×120 s, 14×300 s	
BEH189	April (N/A)	Torso	2 (first scan) 1 (second scan)	i.v. (85.47 MBq) (85.47 MBq)	4×30 s, 4×60 s, 4×120 s, 9×300 s	
BEJ129	April (13.4 kg)	Torso	1 (first run) 2 (second run)	i.v. (106.93 MBq) (111.27 MBq)	4×30 s, 4×60 s, 4×120 s, 9×300 s	
BEH137	Pearl (N/A)	Torso	2 (first scan) 1 (second scan)	i.v. (122.10 MBq) (127.28 MBq)	4×30 s, 4×60 s, 4×120 s, 9×300 s	
BEJ158	Pearl (14 kg)	Brain	1	Oral (341.51 MBq of 1 in 10 mL ethanol and 35 mL water)	5×60 s, 5×180 s, 5×240 s, 6×300 s, 2×600 s	
BEJ196	Missy (N/A)	Brain	1 (first scan)	i.v. (166.13 MBq, 35 min after pretreat with 45 ml water)	12×10 s, 8×30 s, 4×120 s, 14×300 s	
			1 (second scan)	i.v. (185.37 MBq, 35 min after pretreat with 10 ml ethanol and 35 ml of water)		
1: CH_3_^11^CH_2_OH; 2: CH_3_^11^CD_2_OH						

## Results

### Peripheral organ uptake and the deuterium isotope effect

**Figure 1:**
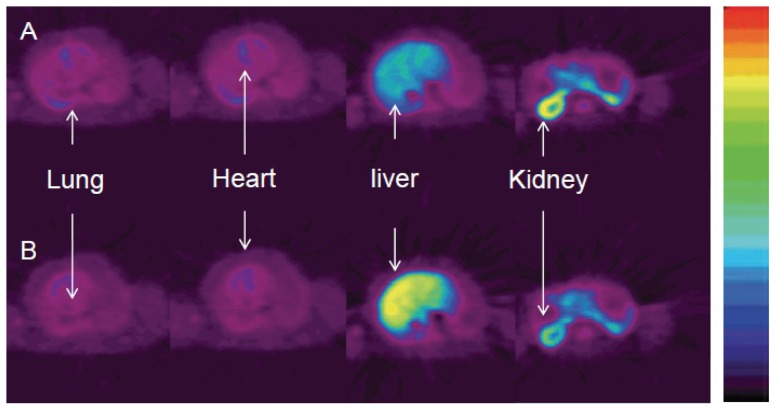
Summed images of tracer 1 (A, top) and tracer 2 (B, bottom) in lung, heart, liver and kidney (from 0 to 60 min) in baboon (April). Tracer 1 had the highest uptake in kidney; tracer 2, had the highest uptake in liver.

**Figure 2:**
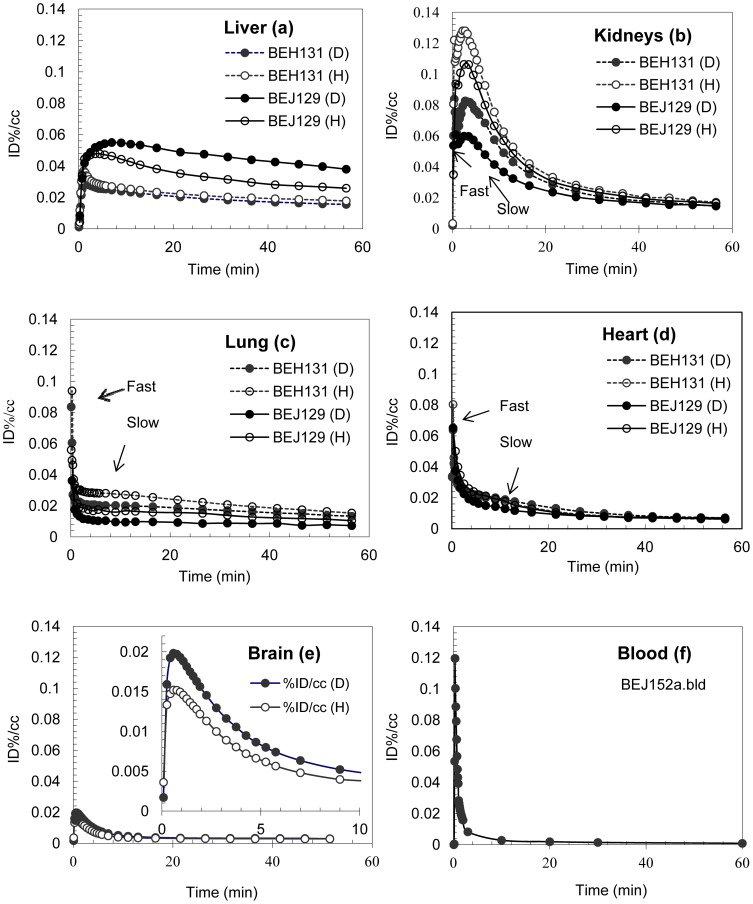
Time activity curves in liver (TAC) (a); kidney (b); lung (c); heart (d) in baboons (April: BEJ129 and Missy: BEH131) and Brain (e) and blood (f) in baboon (Pearl: BEJ152a) of tracers 1 (H) and 2 (D).

Alcohol ADME properties are affected by the genetic predisposition, individual body composition, physical condition, and the environmental factors including past history of alcohol use [33], [34]. Individual variations in alcohol's ADME in the liver, kidneys, lung and heart were also observed between baboons. Therefore, we did not attempt to average the PET data from different baboons for peripheral ADME analysis; instead, we presented results from the two baboons individually, April (BEJ129) and Missy (BEH131) in whom we obtained measurements for the liver, kidneys, lung, and the heart. The heart, lungs, liver, and the kidneys were easily identified from the summed PET images of **1** and **2** (Fig 1). Tracer **1** had the highest uptake in the kidney, tracer **2**, on the other hand, had the highest uptake in the liver. The ADME of **1** and **2** were quantitatively assessed with the following parameters (Table 2) derived from the TAC (Fig. 2a, 2b, 2c, 2d): peak uptake (C_max_); the time at which peak uptake was observed (T_max_); time at which peak uptake was reduced to half of its value (T_1/2_); area under the curve (AUC_60min_) and residual uptake (C_60min_) at end of the scan. Tracers **1** and **2** had different liver uptake patterns in April and Missy (Fig. 2a): the radioactive C-11 from **1** had a lower peak uptake (C_max_ = 0.048% ID . mL^−1^) than that from **2** (C_max_ = 0.055% ID . mL^−1^) in April (Table 2), whereas in Missy peak uptakes from **1** (C_max_ = 0.036% ID . mL^−1^) and **2** (C_max_ = 0.030% ID . mL^−1^) were similar. Both tracers had slow elimination rates (T_1/2_>50 min) from the liver. Tracer **1** had lower liver exposure (AUC_60min_ = 1.92 min . % ID . mL^−1^) than **2** (AUC_60min_ = 2.61 min . % ID . mL^−1^) in April, whereas in Missy liver exposures were similar (AUC_60min_ = 1.24 min . % ID . mL^−1^ for **1** vs 1.11 min . % ID . mL^−1^ for **2**) and the liver exposures from both tracers were lower than that for April. Tracer **1** had a lower residue uptake (C_60min_ = 0.026% ID . mL^−1^) than **2** (C_60min_ = 0.038% ID . mL^−1^) in April and a similar residue uptake (C_60min_ = 0.018% ID . mL^−1^ for **1** and 0.016% ID . mL^−1^ for 2) in Missy.

**Table 2:** Pharmacokinetics assessment of alcohol after *iv* administration to baboon.

Organ	Study	tracer	C_max_ (% ID . mL^−1^)		T_max_ (min)		T_1/2_ (min)		AUC60min min . % ID . mL^−1^	C60min % ID . mL^−1^
liver	BEJ129	**1**	0.048 0.055 0.036 0.030		3.00 7.00 1.25 1.25		>60 >60 52.0 52.0		1.92	0.0260
		**2**							2.61	0.0380
	BEH131	**1**							1.24	0.0180
		**2**							1.11	0.0160
			***faster***	***slower***	***faster***	***Slower***	***Faster***	***slower***		
kidney	BEJ129	**1**	0.094	0.110	0.75	3.00	ND	11.0	1.62	0.0164
		**2**	0.061	0.060	0.75	3.00	ND	14.0	1.05	0.0146
	BEH131	**1**	0.122	0.128	0.42	2.50	ND	11.0	2.35	0.0134
		**2**	0.084	0.083	0.42	2.75	ND	14.0	1.81	0.0109
heart	BEJ129	**1**	0.065	∼0.021	0.25	∼6	1.80	∼20	0.69	0.0060
		**2**	0.065	∼0.015	0.25	∼6	1.00	∼32	0.60	0.0070
	BEH131	**1**	0.080	∼0.021	0.25	∼6	0.50	∼25	0.67	0.0060
		**2**	0.064	∼0.021	0.25	∼6	1.20	∼28	0.74	0.0070
lung	BEJ129	**1**	0.049	0.018	0.25	∼6	0.75	>60	0.82	0.0105
		**2**	0.036	0.011	0.25	∼7	0.75	>60	0.52	0.0073
	BEH131	**1**	0.094	0.029	0.25	∼5	0.42	>60	1.27	0.0154
		**2**	0.084	0.021	0.08	∼5	0.30	>60	1.00	0.0134
brain	BEJ152	**1**	0.015 0.020		0.60 0.60		4.00 4.00		0.23	0.0029
		**2**							0.27	0.0030
blood	BEJ152	**1**	0.118 0.118		0.42 0.42		0.75 0.75		0.21	0.0040
		**2**							0.25	0.0005
brain	BEJ158	**1**	0.038		0.42		2.50		0.31	0.0032
brain	BEJ196	**1**	Brain uptake plateaued at ca. 15 min						0.12	0.0027

C_max_, peak uptake; T_max_, time at which peak uptake was observed; T_1/2_, time at which uptake reduced to half of its peak value; AUC60min: area under time activity curve from 0 to 60 min; C60min residue uptake at end of scan

Tracers **1** and **2** had similar uptake patterns for April and Missy in the kidney, heart, and the lung (Fig. 2b, 2c, 2d). A faster uptake-elimination phase (T_max_ = 0.08 to 0.75 min; T_1/2_ = 0.3 to 1.8 min) was followed by a slower uptake-elimination phase for **1** and **2** in these organs for both baboons (Table 2). During the faster uptake-elimination phase, the alcohol simply perfused through the organ along with the blood flow with minimal biochemical tissue interactions; we define this as the physical uptake-elimination phase. In the slower uptake-elimination phase, alcohol and its metabolites interact with tissues biochemically. Information from the alcohol's metabolism can be derived from the slower uptake and elimination phase. Tracer **1** had higher uptake, faster elimination, and higher residue uptake than **2** in the kidneys in each baboon. The elimination of both tracers from the heart was similar and in the lungs it was higher for **2** than **1** with T_1/2_ from 20 to 32 min in lung and over 60 min in heart.

### Alcohol brain uptake and deuterium isotope effect

**Figure 3:**
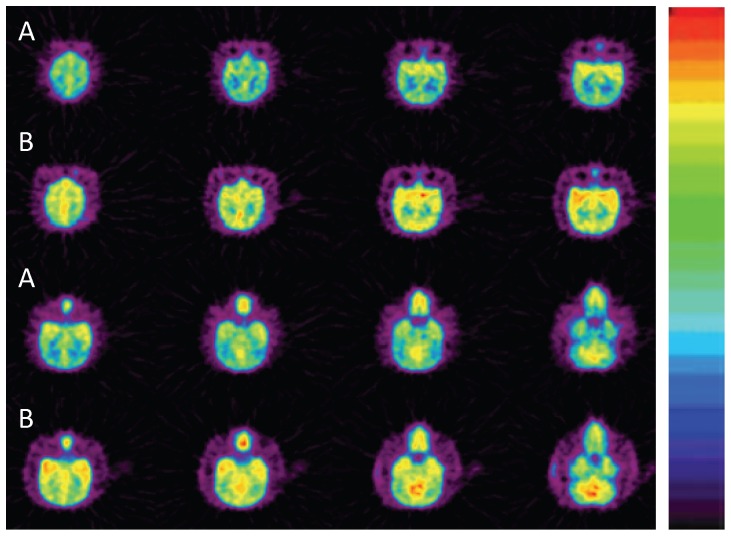
Summed images of tracers 1 (A) and 2 (B) in the brain of Pearl (from 0 to 10 min), alcohol distributed in all brain regions. Tracer 1 showed higher uptake in striatum and cerebellum; and tracer 2 in striatum, thalamus, cerebellum, and cingulate. Tracer 2 had consistently higher uptake than 1 across all brain regions.

**Figure 4:**
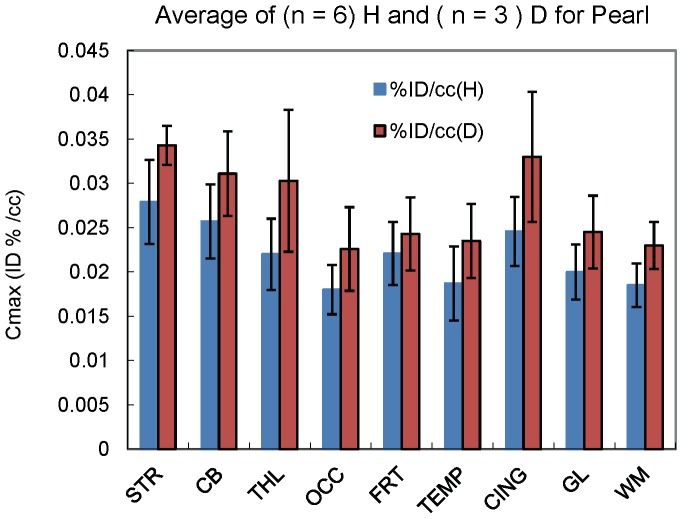
Brain uptake (C_max_) of tracers 1 (H) and 2 (D) in baboon (Pear) in different brain region. STR: Striatum; CB: Cerebellum; THL: Thalamus; OCC: Occipital cortex; FRT: Frontal cortex; TEMP: Temporal cortex; CING: Cingulate gyrus; GL: Global; WM: White matter. Tracer 2 shows consistent higher uptake than 1 in all brain regions.

To avoid intersubject variability, alcohol's brain uptake were studied in the same baboon (Pearl) with tracers **1**and **2**. The metabolic effect on brain uptake was assess by observed deuterium isotope effect. A total of nine studies were conducted, six with **1** and three with **2**. Alcohol was distributed in most of the brain regions, but showed higher concentration in striatum (C_max_ = 0.028% ID . mL^−1^) and cerebellum (C_max_ = 0.026% ID . mL^−1^) for **1** and in striatum (C_max_ = 0.034% ID . mL^−1^), cingulate (C_max_ = 0.033% ID . mL^−1^), cerebellum (C_max_ = 0.031% ID . mL^−1^), and thalamus (C_max_ = 0.030% ID . mL^−1^) for 2 (Fig 3). The C_max_ ratio of brain to blood obtained during the first 3 minutes is 0.13 for 1 and 0.18 for **2**. Tracer **2** had consistently higher peak uptake (C_max_) than **1** in all brain regions studied (Fig 4). The C_max_ ratio for the peak uptake of **2** to **1** was highest in thalamus (C_max_(**2**)/C_max_(**1**) = 1.38) and cingulate (C_max_(**2**)/C_max_(**1**) = 1.34) and lowest in frontal cortex (C_max_(**2**)/C_max_(**1**) = 1.10) (Fig S1).

**Figure 5:**
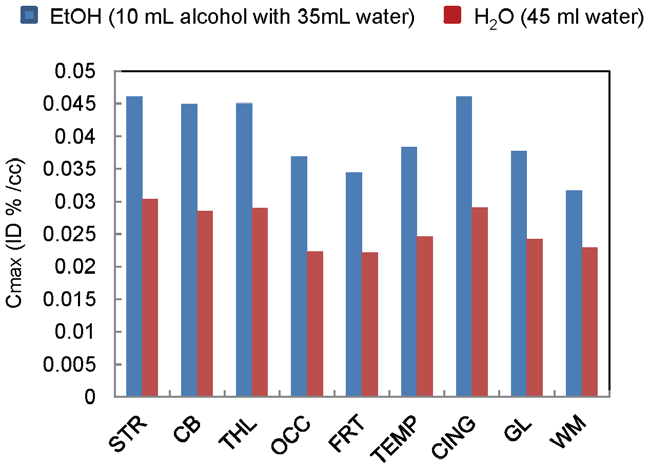
Brain uptake (C_max_) of tracer 1 in baboon (Missy) in different brain region. STR: Striatum; CB: Cerebellum; THL: Thalamus; OCC: Occipital cortex; FRT: Frontal cortex; TEMP: Temporal cortex; CING: Cingulate gyrus; GL: Global; WM: White matter. When baboon was pretreated with water (45 ml) or alcohol (22% ABV, 45 ml).

**Figure 6:**
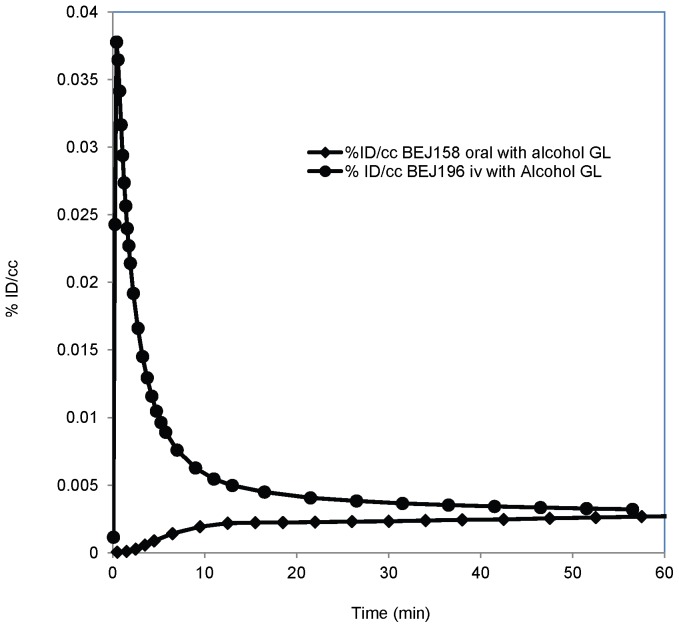
Time activity curves for the global brain uptake when tracer 1 was administered *iv* 35 min after baboon was pretreated with alcohol (22% ABV, 45 ml), and when tracer 1 was administered orally with alcohol (22% ABV, 45 ml). The brain exposure (AUC_60min_) was much higher when tracer 1 was administered iv. GL: Global

To further study the effect of metabolism on alcohol's brain uptake we conducted a pair of sequential studies in the same baboon (Missy) with 1 (BEJ196): the first scan after pretreatment with water (45 ml), and the second one, 35 minutes after pretreatment with a pharmacological relevant dose (5.6 g/kg) of alcohol (22% alcohol by volume (ABV), 45 mL). Pretreatment with alcohol significantly increased the brain uptake of alcohol in all brain regions (Fig 5). To evaluate the effect of the first pass metabolism and intestine permeability on alcohol brain uptake, a pharmacological relevant dose of alcohol spiked with 1 (341.51 MBq) was administered to Pearl (BEJ158) orally, the brain alcohol uptake plateaued at approximately 15 min in all brain regions and remained at that level throughout the 60 minutes of the study (Fig 6). The study BEJ158 (oral 1 in a pharmacological dose of alcohol) was compared with the BEJ196 second scan (intravenous 1 after an oral pharmacological dose of alcohol) (Fig 6), and 1 was found to have a much higher brain exposure and peak uptake after *iv* than after oral administration. However at 50 minutes the brain concentration of alcohol was similar for *iv* and oral administration.

## Discussion and Conclusions

Ethanol is metabolized to acetaldehyde in the body through three major pathways: (1) an alcohol dehydrogenase (ADH) pathway, which accounts for over 85% of ethanol's oxidation and has a deuterium isotope effect of 3 and is pH (pH = 7) and coenzyme dependent (coenzyme NAD+) [35], ADH pathway is reversible in *vivo* and a reversible-ADH pathway converts acetaldehyde back to alcohol and causes the hydrogen for deuterium exchange (CH_3_CD_2_OH to CH_3_CHDOH and CH_3_CDHOH to CH_3_CH_2_OH) eliminating the deuterium isotope effect [29]; (2) catalase pathway, which eliminates about 2% ethanol and has a deuterium isotope effect of 1.9 determined from rat and ox liver catalase [36], [37]; and (3) the microsomal ethanol-oxidizing system (MEOS) is a minor metabolic pathway in healthy humans, but in alcoholics it can account for up to 10% [38] of ethanol's elimination in the liver and has a deuterium isotope effect from 3.6 to 5.2 [39]. Acetaldehyde is then oxidized to acetate by acetaldehyde dehydrogenase (ALDH) and it has a deuterium isotope effect of 2.8 [40].

The liver is by far the most important organ for ethanol's elimination and it contains almost all the ethanol metabolic enzymes. In healthy animals or humans, hepatic alcohol metabolism is responsible for over 95% of ethanol's oxidation. Individual genetic makeup and environmental condition could alter the contribution of individual metabolism pathways to the overall alcohol metabolism [41]. The two baboons in whom we studied, the liver differed in their metabolism of **1** and **2** as evidenced by their liver TAC profiles (Fig 2a), which represent hepatic uptake and elimination kinetics for all C-11 labeled species derived from C-11 labeled alcohol including C-11 labeled alcohol, acetaldehyde, acetate, carbon dioxide, and higher molecular metabolites. The hepatic TAC profiles for **1** and **2** were different in April (higher for **2** than **1**), but similar in Missy. The liver TAC patterns for **1** and **2** could be used as a biomarker to assess the contributions from different alcohol metabolism pathways to overall hepatic alcohol metabolism, the reliability of such marker would have to be confirmed by enzyme inhibition and in a larger sample size.

Studies done in *ex-vivo* isolated renal tissue (cortex and tubules) from baboons showed that the reversible ADH pathway is present in the kidney. In the kidney, acetaldehyde can be metabolized at a high rate and in a dose dependent manner and converted to ethanol, acetate and carbon dioxide; at acetaldehyde concentration from 1 mM to 20 mM the major product is acetate and at higher acetaldehyde concentration the major product is ethanol [20], [42]. Our PET images show that in the kidneys ethanol's metabolism mainly took place in the cortex (Fig 1). The activity derived from **1** was eliminated consistently faster (T_1/2_ = 11 min) than **2** (T_1/2_ = 14 min) in both baboons (Table 2), this isotope effect may indicate the contribution of oxidation of acetaldehyde (CH_3_CHO from **1** or CH_3_CDO from **2**) to acetate. The slower elimination of **2** than **1** in both baboons may suggest the transformation of acetaldehyde into acetate in the kidneys, which is supported by the well-recognized role that kidneys have in the detoxification of alcohol from the body [20].

Studies on lung slices from rats and dogs showed that alcohol dehydrogenase in pulmonary tissue can metabolize ethanol in a bicarbonate buffer by sulfoconjugation [19], [43] but in human this is likely to be limited by the substrate availability. In the slower uptake elimination phase, the time-activity-curve reached a steady state in the lungs, which could be attributed to ^11^CO_2_ elimination. The expiration of ^11^CO_2_ from ethanol showed that it reached a steady state shortly after intravenous injection [44]. The overall slower metabolism of **2** would therefore result in a lower blood concentration of ^11^CO_2_, and less carbon-11 exchange in the lungs through ^11^CO_2_ expiration, which would account for the lower residual activity (C_60min_) for **2** than for **1** (Table 2).

Like the lungs, the heart is not directly involved in oxidative metabolism of ethanol. The overall heart exposure (AUC_60min_) and heart residue uptake (C_60min_) for tracers **1** and **2** was similar for both baboons (Table 2). Ethanol in the heart can be converted into fatty acid ethyl ester (FAEE) by FAEE synthase enzyme, which is detrimental to heart muscles [45]. No carbon-hydrogen bond is broken or made in this process, thus the deuterium isotope effect is not expected.

Metabolism of ethanol in the peripheral organs has a significant impact on the uptake of alcohol by the brain. The up-to-date consensus has been that the acetaldehyde that is peripherally derived does not penetrate the blood brain barrier in any significant amount [1]. The uptake of acetate by the brain is low [46] but it increases during alcohol intoxication (Volkow et al unpublished). Thus the radioactivity in the brain for both tracers most likely reflected ethanol's brain uptake with some contribution from acetate particularly when given concomitantly with pharmacological doses of alcohol. The slower metabolism of **2** resulted in a higher plasma alcohol concentration, which would account for its higher brain peak uptake (Fig 4). When the baboon was pretreated with a pharmacological dose of alcohol, the alcohol metabolizing enzymes (ie ADH and catalase) may have been saturated resulting in the elevated tracer (**1**) blood concentrations and higher brain uptake of **1** than when pretreated with water. However, the higher blood flow [47] and the higher acetate concentration in plasma under the influence of alcohol (Volkow et al unpublished) may have also contributed to the initial higher brain uptake of **1**. This added to our findings of much lower brain uptake after oral alcohol administration, which exposes alcohol to the first pass hepatic metabolism, than after intravenous administration, and the higher brain uptake for **2**, which has lower peripheral metabolism than **1**, provides further evidence that peripheral metabolism of alcohol influences the uptake of alcohol in the brain. To the extent that there are significant differences in the rate of alcohol metabolism between individuals, including greater metabolism in alcoholics than controls and greater metabolism in males than females [48]. This would contribute to the differences in the sensitivity to alcohol's psychoactive effects.

Alcohol distributed throughout the brain but there was a greater uptake and accumulation in the cerebellum and striatum for both tracers but also higher uptake in cingulate and thalamus for **2** (Fig 3). This is consistent with prior studies in the rodent brain that showed the highest alcohol concentration in the striatum [49] and with a prior PET study in cymologous monkeys that reported higher [C-11]ethanol uptake in subcortical brain regions [50]. The higher uptake in striatum would underlie its rewarding effects, which are mediated in part by its effects in ventral striatum [51], [52]. On the other hand the high accumulation of alcohol or its metabolites in the cerebellum is consistent with findings that the cerebellum is particularly sensitive to the decreases in brain glucose metabolism after acute alcohol administration [53] and could underlie the motor incoordination observed during intoxication [54].

The peak uptake (C_max_) ratio of brain to blood is 0.13 for **1** and 0.18 for **2**, which occurs within the first 3 minutes after its administration most likely reflects the high concentration of alcohol in blood (Fig S2). However after 3 minutes the blood to brain ratio reaches 1 and then slowly decreases, which is consistent with our findings from MRS in humans showing blood to brain ratios for alcohol around 1 or lower [25]. Pharmacokinetic studies of alcohol in the rat brain after an intraperitoneal injection showed that its uptake in the first 5–10 minutes reflects primarily absorption after which it reflects a combination of absorption, metabolism, elimination and water/fat equilibration [55]. Our findings of a C_max_ ratio of brain to blood obtained during the first 3 minutes of 0.13 for **1** and 0.18 for **2** and its rapid equilibration to brain to blood ratios of 1 are also consistent with its uptake initially being driven by its absorption and subsequently driven by a combination of absorption, metabolism and clearance.

The first pass alcohol metabolism has a significant impact on the alcohol blood concentration [56]. Our PET data shows that the area under the curve (AUC_60min_) after intravenous administration is significantly higher than that after oral administration (Table 2, BEJ158 and BEJ196) (Fig 6) indicating that gastric absorption and the first pass alcohol metabolism significantly reduces alcohol overall brain's exposure and delays its arrival to the brain.

Limitation from our studies includes the fact that PET measures the overall concentration of C-11 activity within the tissue but cannot distinguish between alcohol and its metabolites. Also because of the complexity and high cost of the studies, only a small group of animals were used in the measurments that precludes us from addressing the intersubject variability of alcohol's ADME in primates.

In conclusion, our studies demonstrate the value of using the deuterium isotope effect and PET to investigate the ethanol ADME properties in the liver, kidney, lung, heart, and the brain. This study corroborates alcohol metabolism by the liver and kidneys, and demonstrated that peripheral alcohol metabolism has significant impact on alcohol's brain bioavailability. These findings sets the stage for future studies of alcohol in humans to investigate how genetics, gender and alcohol abuse affect alcohol's ADME in the various organs, including brain and its relationship to intoxication and medical consequences.

## Supporting Information

Figure S1 **Brain uptake ratio (C_max_) of tracer 2 (D) to 1 (H) in baboon (Pear) in different brain region.** STR: Striatum; CB: Cerebellum; THL: Thalamus; OCC: Occipital cortex; FRT: Frontal cortex; TEMP: Temporal cortex; CING: Cingulate gyrus; GL: Global; WM: White matter. The ratio was highest in thalamus and cingulate gyrus and lowest in frontal cortex
(TIFF)

Figure S2 **The ratio of tracer (1) blood concentration (C(blood)) to tracer brain concentration (C(brain)) vs time in baboon (Pear).**
(TIFF)
