# Correction: Alcohol ADME in Primates Studied with Positron Emission Tomography

**DOI:** 10.1371/annotation/ad777b60-57d0-4366-a15e-3bab9a77cd6d

**Published:** 2013-05-17

**Authors:** Zizhong Li, Youwen Xu, Don Warner, Nora D. Volkow

There were errors in Table 2. The correct version of the table is available here: 

**Figure:**
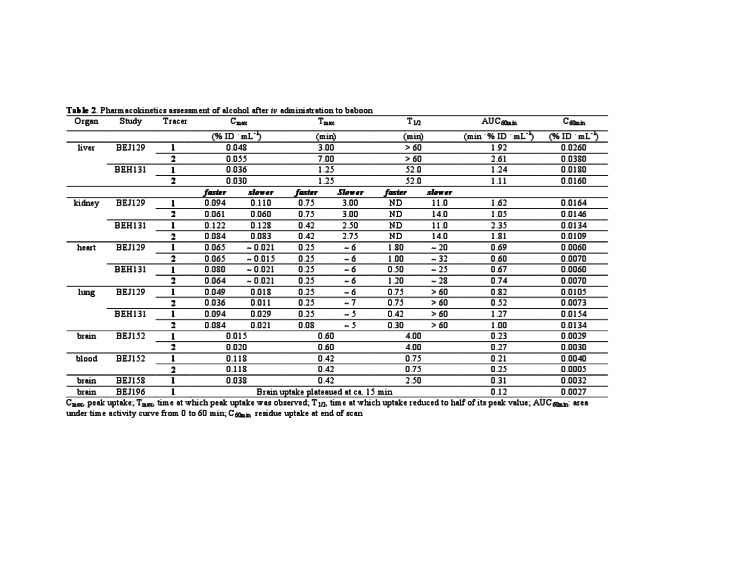


In addition, there were multiple instances in the article where "Volkow et al unpublished" was referenced. The article has been published, and the full reference is:

Volkow ND, Kim S, Wang GJ, Alexoff D, Logan J, et al. (2012) Acute alcohol intoxication decreases glucose metabolism but increases acetate uptake in the human brain. Neuroimage. 

