# Supplementary figures and images for: Alcohol ADME in Primates Studied with Positron Emission Tomography
Source: PLoS One. 2012 Oct 1;7(10):e46676. doi: 10.1371/journal.pone.0046676 (PMC3462207; doi:10.1371/journal.pone.0046676)

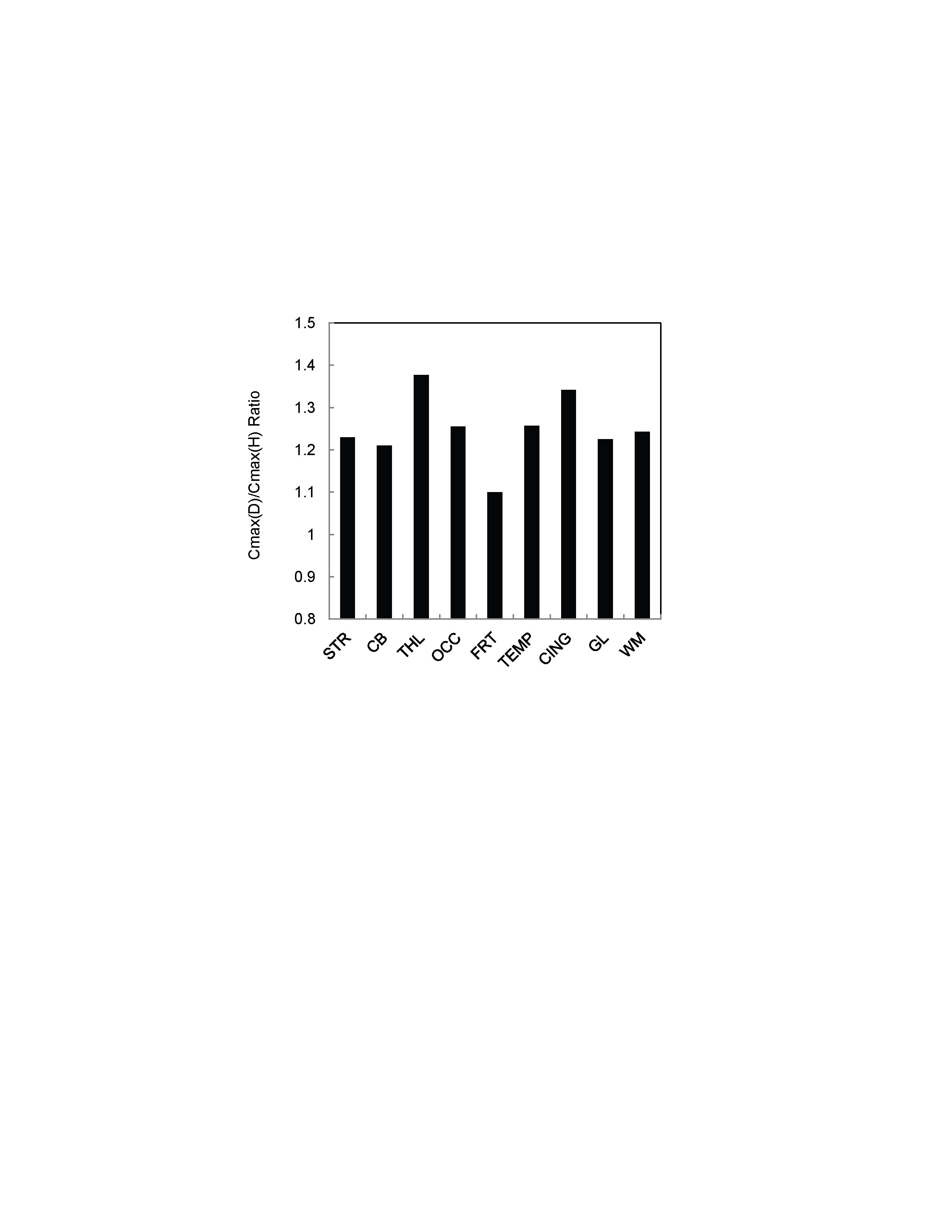

Supplement: Figure S1 — Brain uptake ratio (Cmax) of tracer 2 (D) to 1 (H) in baboon (Pear) in different brain region. STR: Striatum; CB: Cerebellum; THL: Thalamus; OCC: Occipital cortex; FRT: Frontal cortex; TEMP: Temporal cortex; CING: Cingulate gyrus; GL: Global; WM: White matter. The ratio was highest in thalamus and cingulate gyrus and lowest in frontal cortex (TIFF) [file pone.0046676.s001.tiff]

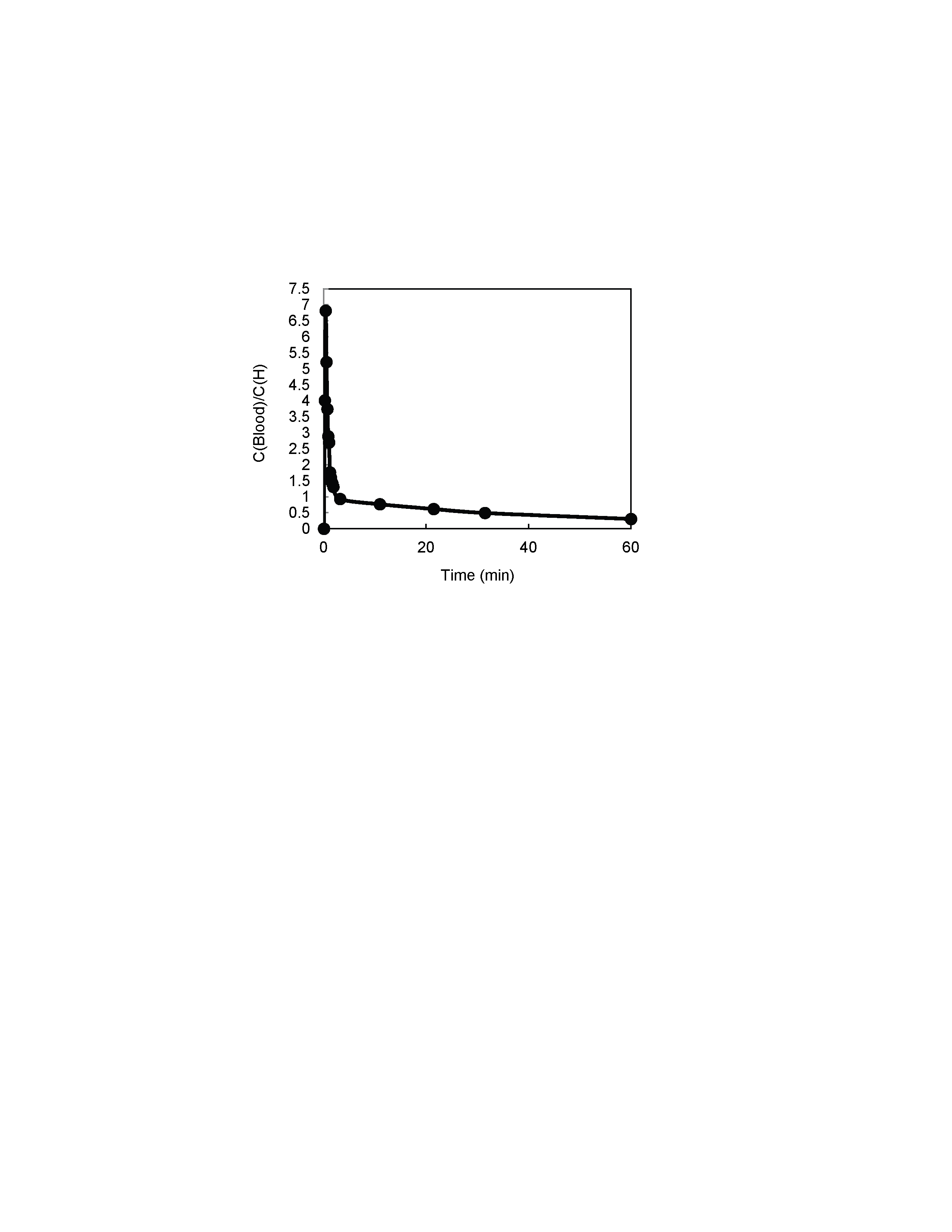

Supplement: Figure S2 — The ratio of tracer (1) blood concentration (C(blood)) to tracer brain concentration (C(brain)) vs time in baboon (Pear). (TIFF) [file pone.0046676.s002.tiff]
